# Gene Expression Comparison between the Lymph Node-Positive and -Negative Reveals a Peculiar Immune Microenvironment Signature and a Theranostic Role for WNT Targeting in Pancreatic Ductal Adenocarcinoma: A Pilot Study

**DOI:** 10.3390/cancers11070942

**Published:** 2019-07-04

**Authors:** Antonella Argentiero, Simona De Summa, Roberta Di Fonte, Rosa Maria Iacobazzi, Letizia Porcelli, Matteo Da Vià, Oronzo Brunetti, Amalia Azzariti, Nicola Silvestris, Antonio Giovanni Solimando

**Affiliations:** 1 Medical Oncology Unit, IRCCS Cancer Institute “Giovanni Paolo II” of Bari, 70124 Bari, Italy; 2 Molecular Diagnostics and Pharmacogenetics Unit, IRCCS Cancer Institute “Giovanni Paolo II”, 70124 Bari, Italy; 3 Experimental Pharmacology Laboratory, IRCCS Cancer Institute “Giovanni Paolo II”, 70124 Bari, Italy; 4 Department of Internal Medicine II, Interdisciplinary Center for Clinical Research Laboratory, University Hospital of Würzburg, 97080 Würzburg, Germany; 5 Medical Oncology Unit, The Hospital Mons. R. Dimiccoli, 76121 Barletta (Bat), Italy; 6 Scientific Direction, IRCCS Cancer Institute “Giovanni Paolo II”, 70124 Bari, Italy; 7 Department of Biomedical Sciences and Human Oncology, Section of Internal Medicine ‘G. Baccelli’, University of Bari Medical School, 70124 Bari, Italy

**Keywords:** pancreatic cancer, PDAC, lymph node metastases, WNT, dendritic cells, M2 macrophages, XAV-939, ANGPTL4, MST-1, tumor immune microenvironment

## Abstract

Over the past several years there has been much debate with regards to the prognostic and clinical significance of pancreatic ductal adenocarcinoma (PDAC) with lymph nodes metastasis. The PDAC gene expression knowledge and the biologic alterations underlying the lymph node involvement convey a clinical implication in dealing with the theranostic window. To this end, we provide an original bioinformatic dissection of the gene expression differences of PDAC according to the nodal involvement from a large public available dataset. Comprehensive transcriptomic analysis from 143 RNA-seq patient’s derived samples indicated that WNT increased activation and a peculiar immune microenvironment identify subjects with nodal involvement. In frame of this thinking, we validated the WNT pathway role in increasing the likelihood of lymphatic dissemination in vitro. Moreover, we demonstrated for the first time in a PDAC model the potential therapeutic window that XAV-939—a specific WNT pathway inhibitor—has in re-educating a tumor-permissive immune system. Finally, we outline the potential implication on bystander molecular drivers exerted by WNT molecular inhibition, providing a picture of the proteomic oncogenic landscape changes elicited by XAV-939 on PDAC cells and their clinical implication. Our findings hold the promise to identify novel immune-based therapeutic strategies targeting WNT to enhance PDAC cytotoxicity and restore anti-PDAC immunity in node-positive disease.

## 1. Introduction

According to international guidelines, regional lymph node (LN) metastases are not considered a surgery contraindication for the treatment of resectable pancreatic ductal adenocarcinoma (PDAC) [1,2]. Nevertheless, the presence of LN metastases as well as the number of positive LNs strongly impact the PDAC outcome.

Recently, the International Association of Pancreatology proposed to include the suspicion of regional LN metastases in the criteria defining borderline resectable-PDAC [3]. This frame of thinking affects a further selection of patients eligible for up-front surgery with the aim to reduce surgical morbidity and mortality in poor prognosis patients. The scientific community is, in fact, now involved in identifying prognostic factors for patient management and clinical decision-making [4].

Recent progresses in molecular profiling of PDAC helped in achieving a better stratification of different clinical phenotypes highlighting a possible role of genetic signatures in the LN metastatic process [5,6,7,8]. Lymphatic dissemination depends on the interaction among cancer cells, the tumor immune microenvironment and the premetastatic niche. However, to date, no gene signature has been dissected in order to find a lymphatic-related metastasis driver in PDAC [9].

Therefore, a better understanding of the underlying biology and could be useful in predicting high risk feature for LN involvement and in identifying a subgroup of patients with poor prognosis that could benefit from neoadjuvant targeted therapeutic strategies.

To this end, we interrogated the ICGC data portal and molecularly analyzed RNA-seq from 143 PDAC patients and shed more light onto the definition of lymph nodes metastases biology. Next, we identified a distinctive molecular gene expression signature, characteristic for LN positive PDAC patients, translating our original data into a specific clinical scenario. We validated a novel potential approach in vitro, to better tailor the tumor directed target therapy and to re-educate a permissive cancer milieu that potentially offered the possibility of a novel approach for PDAC patients.

## 2. Results

### 2.1. Gene Expression Analysis Comparing PDAC Patient-Derived Specimen Identified a Characteristic Biological Signature Depending on Lymph Node Status

**Figure 1:**
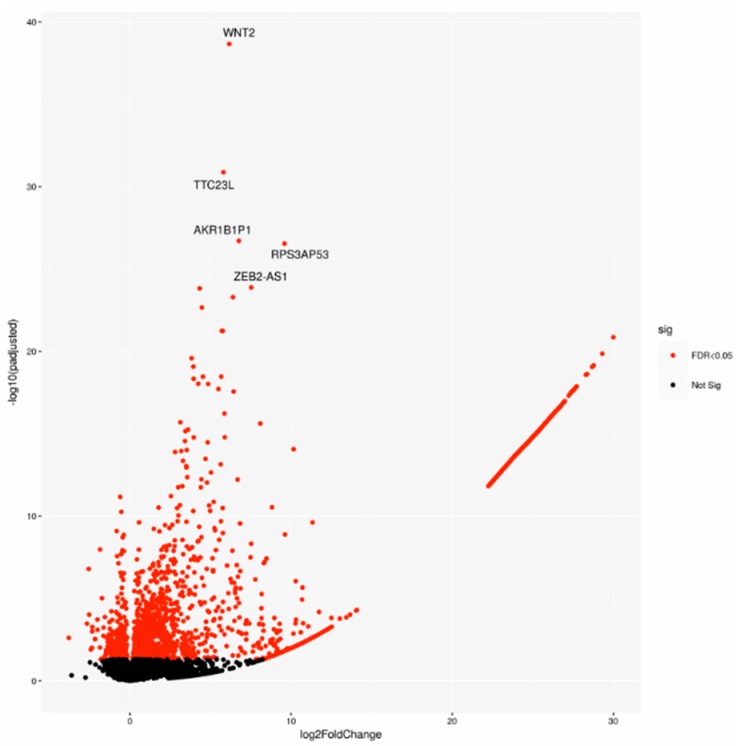
Volcano plot of differentially expressed genes (DEGs), highlighting the top 5 DEGs. Sig: significant.

A cohort including 143 cases was chosen on the basis of the pathological stage from the PACA-CA study. In detail, 64 LN-positive (N^pos^) patients and 79 LN-negative (N^neg^) samples underwent differential gene expression analysis. Two-thousand-one-hundred-and-forty-seven significantly deregulated genes (DEGs) were identified (Figure 1), indicating a large modulation of gene expression in relation to lymph node status.

**Figure 2:**
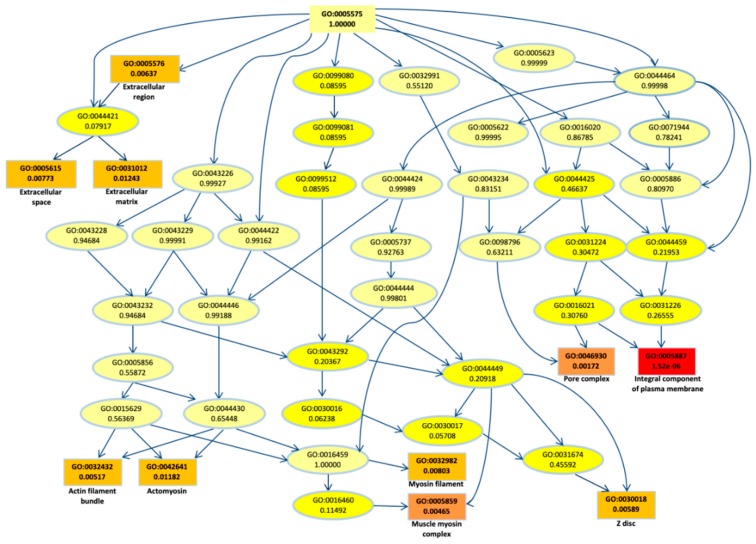
Gene ontology (GO) graph related to the top 10 most enriched terms. Significant GO accession codes are reported in rectangles and statistical significance is shown in color scale that ranges from yellow to red (low to high significance). At the right side, the descriptions related to the significant terms are reported.

To better understand their biological role, first we performed a gene ontology (GO) enrichment analysis for the DEGs. The top most significant 10 GO terms were located on the GO graph (Figure 2) in order to visualize the relationship between them. In particular, we observed term related to myosin or actomyosin or to membrane component, with “integral component of plasm membrane” term as the most significant (*p*-value < 10^−5^).

### 2.2. Biological Network and Gene Set Enrichment Analysis of Database of Essential Genes

**Figure 3:**
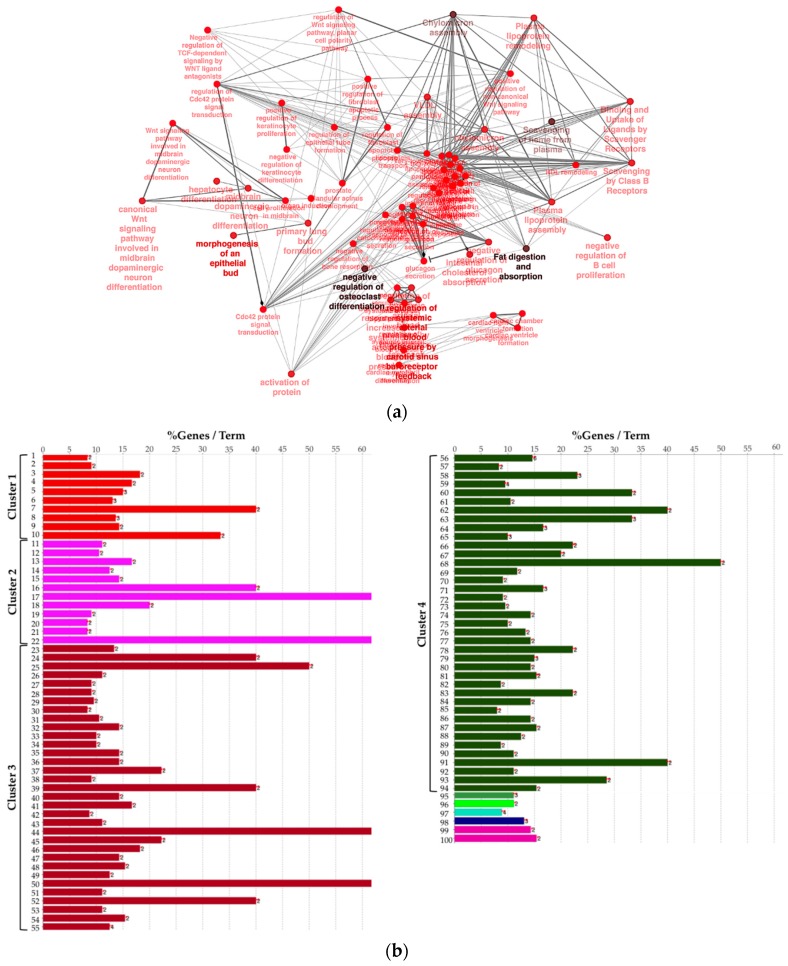
(**a**) Biological network including significant enriched terms related to KEGG, GO Biological Process, Reactome pathway, and GO immune system process databases. (**b**) Bar plot showing the grouping of the terms showed in the network, see text and Supplementary File 1 for details.

Next, to provide a more comprehensive functional picture, we created a biological network of DEG by using the Cytoscape app ClueGO. In the Figure 3a, the network is represented radially, showing a large nucleus in the center including terms like “negative regulation of heterotypic cell–cell adhesion” and “negative regulation of cytokine secretion involved in immune response”. WNT pathway-related terms were also found to be significant and were therefore included in the biological network. In order to summarize conveniently, in Figure 3b the list of presented significant enriched terms from the same network is grouped by “cluster” of terms.

**Figure 4:**
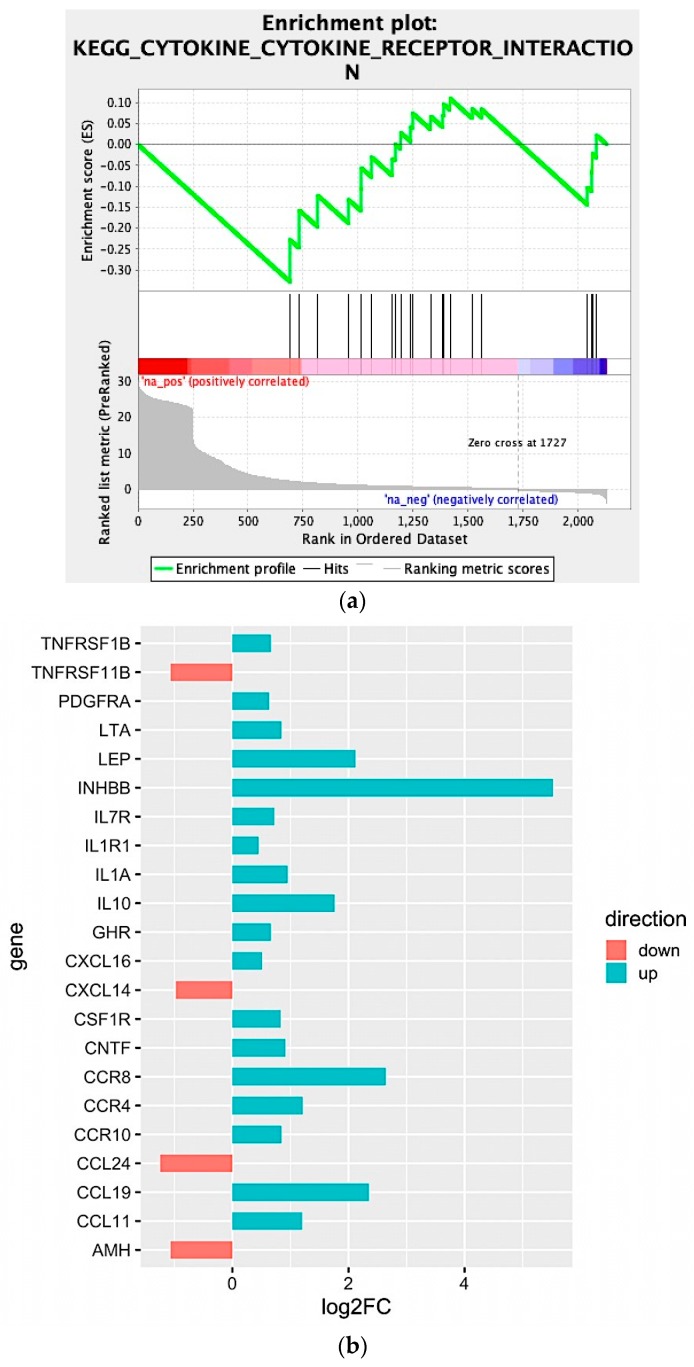
(**a**) Enrichment score graph of GSEA analysis. (**b**) Genes mostly contributing to the enrichment of “KEGG_CYTOKINE_CYTOKINE_RECEPTOR_INTERACTION” and their log2fold-change values.

We observed that four large clusters of terms are present in the biological network, in particular one including the WNT signaling pathway and terms related to epithelial modelling (light and dark red) and the green group with terms including cytokine regulation and immune response. Interestingly, in almost every group a term related to WNT signaling is included, highlighting a pleiotropic function (the list of significant terms and the related genes involved in the enrichment are reported in Supplementary File 1). The ranked list of DEGs was then used to perform the Gene Set Enrichment Analysis (GSEA) analysis. In particular, we found as significantly enriched Kyoto Encyclopedia of Genes and Genomes (KEGG) gene set the “cytokine-cytokine receptor interaction” (q-value < 0.25) (Figure 4a). The core enrichment genes are showed in Figure 4b, with their log2fold-change values. Remarkably, one of the most significant enrichment pointed out the INHBB, a member of the transforming growth member of the TGF-β (transforming growth factor-beta) superfamily.

Moreover, CCR8, CCL19, IL10, IL1A, IL7R, IL1R1, and LEP were also upregulated within the N^pos^ patient’s subgroup. Conversely, we found that CCL24, TNFRSF11B, AMH, and CXCL14 were downregulated. 

### 2.3. In Silico Microenvironmental Composition Estimation

**Figure 5:**
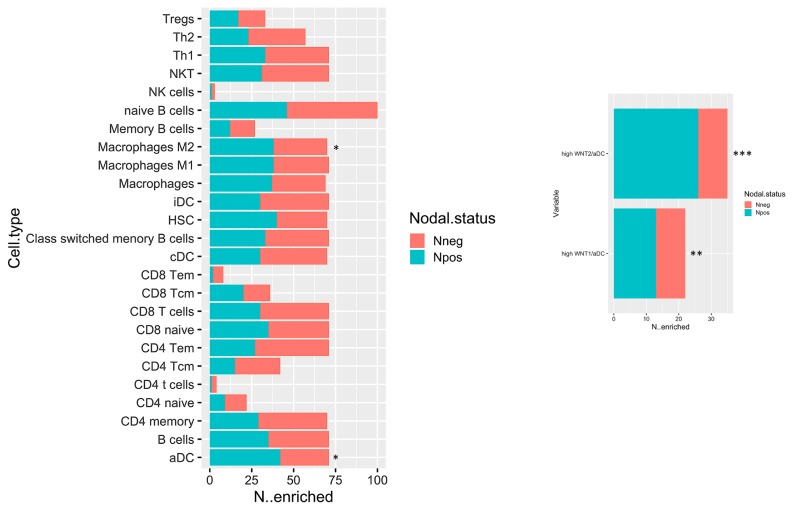
Bar plot displaying the number of cases which have enrichment value above the selected cut-off (median of enrichment scores) relative to N^pos^ and N^neg^ cases. * *p* < 0.05; ** *p* < 0.05; and *** *p* < 0.001.

The biological network results prompt a deeper immune microenvironment characterization. Therefore, we deconvolved Gene expression data to obtain an estimation of the microenvironmental composition. Thus, a normalized gene expression matrix was uploaded to the xCell webtool using a 64-cell type signature matrix to estimate cytotypes enrichment scores. Interestingly, we found that the number of enriched N^pos^ PDAC in M2 macrophages and activated dendritic cells (aDCs) is significantly higher than that of the N^neg^ samples (*p*-value < 0.05) (Figure 5, left panel). Additionally, despite the fact that it is not statistically significant, we found a trend that showed decreased enrichment in T CD8/CD4 cells in the N^pos^ PDAC group. With the goal of immune microenvironment characterization in mind, we next investigated the T cell subpopulation, confirming a decreased enrichment in the effector memory population (both CD8 and CD4 Tem). Additional findings pinpointed an enrichment in hematopoietic stem cells (HSC) in the N^pos^ PDAC patients derived samples. Of note, aDC enrichment correlated with WNT^high^ subgroup in N^pos^ subjects (Figure 5, right panel).

The subset of N^pos^ patients has been stratified according to aDC enrichment scores. Survival estimations in terms of overall survival (OS) and relapse-free survival (RFS) have been calculated. Despite the fact that it is not statistically significant, it can be observed that patients with high aDC enrichment have a poor outcome both for OS and RFS (Supplementary Figure S1).

### 2.4. WNT Inhibition Enhances Pancreatic Cancer Cell Immune-Mediated Killing In Vitro

The increased expression of WNT is conventionally related to the establishment of immunotolerance [10,11,12]. Given that an increased accumulation of the activated DC and M2 macrophages parallels the decreased enrichment of effectors T cells within the microenvironment of the tumor in N^pos^ PDAC patients as respect to the N^neg^ ones (Figure 5, left panel), we next investigated if WNT could represent a promising pharmacological target for activating the immune response. The in vitro validation of this hypothesis was carried out in two PDAC cell lines: MIAPaCa-2 and PANC-1 cells. The first cell line derived from the pancreas adenocarcinoma of a 65-year-old man in whom the tumor involved the body and tail of the pancreas and had infiltrated the periaortic area [13]. The second cell line derived from a 56-year-old male with a pancreatic head adenocarcinoma which invaded the duodenal wall and had metastases in one peripancreatic lymph node [13]. Thus, MIAPaCa-2 and PANC-1 cells are representative of N^neg^ and N^pos^ PDAC. The inhibition of WNT/β-catenin pathway was obtained with XAV-939, a tankyrase (TNKS) inhibitor which antagonizes WNT signaling via stimulation of β-catenin degradation. We explored this hypothesis using the short-term co-cultures of PDAC cells with peripheral blood mononuclear cells (PBMCs) to study their active interaction even in the presence of XAV-939. Furthermore, more than examining their ability to acquire, process, and present to T lymphocytes tumor-derived antigens driving the differentiation of naive T cells into activated tumor-specific effector lymphocytes, we investigated whether DC exerts also a direct cytotoxic effect against tumors [14]. With this aim in mind, we used short-term co-cultures of PDAC cells with purified mDC even in the presence of XAV-939.

#### 2.4.1. XAV-939 Restrains PDAC Cells Tumor Functions, Exerts Cytotoxic Effect, and Halts Cancer-Migration Potential

**Figure 6:**
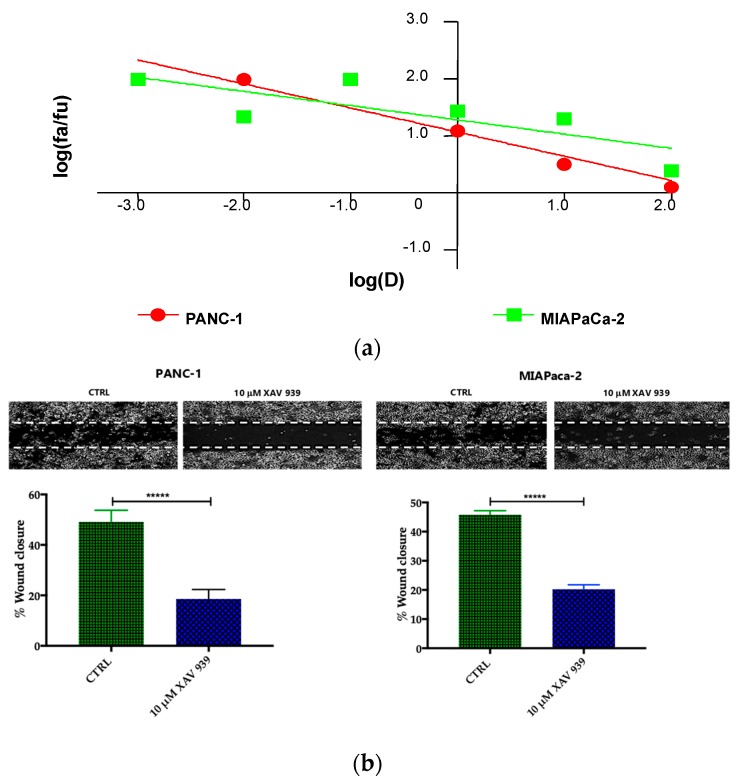

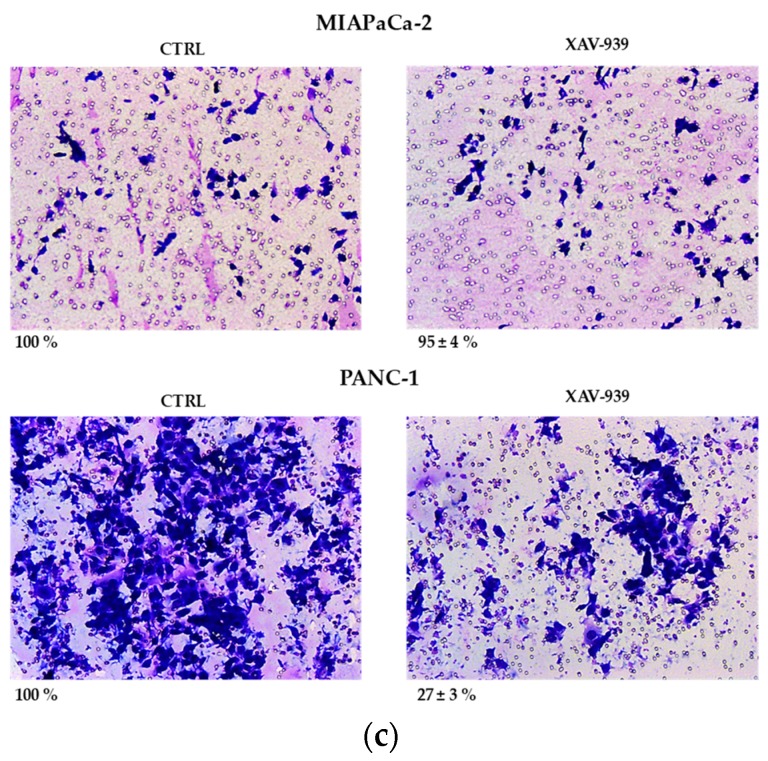
(**a**) Median effect plot showing the inhibition of cell proliferation of PANC-1 and MIAPaCa-2 cells after 48 h treatment with XAV-939 (0.001–100 µM) as log (fraction affected/fraction unaffected) vs. log(dose). (**b**) Effect of XAV-939 on PDAC cells migration was assessed in wound healing assay: XAV-939 treatment restrains PDAC migration function in vitro. WNT-β-catenin inhibition reduced the capacity of tumor cell migration. Differences in wound closure were assessed by ImageJ Lab 1.51 Software. Graph bars represent the percentage of surface area in three independent experiments. Values are expressed as mean ± SD of three independent experiments; **** *p*-value < 0.0001, by *t*-test. (**c**) The invasion of PANC-1 and MIAPaCa-2 cells was performed with or without 10 µM XAV-939 by Boyden chambers assay showing the high capability of the drug in reducing PANC-1 cell invasion.

Preliminary to the killing experiments, we evaluated, after 48 h of incubation the cytotoxicity of XAV-939 on two PDAC cell lines—PANC-1 and MIAPaCa-2—by CCK-8 proliferation assay. The drug exerted higher anti-tumor activity in PANC-1 than in MIAPaCa-2 cells. The highest tested drug concentration (100 µM), the % cell viabilities observed were 56 ± 3% and 71 ± 5% in PANC-1 and MIAPaCa-2, respectively and the IC_50_ values extrapolated with Calcusyn software were 340 µM for PANC-1 and >1000 µM for MIAPaCa-2 cells. The cell proliferation (%)/dose plots of the mean of three different experiments are shown in Figure 6a. The remarkably high IC50s are in agreement with data reported in literature [15], but are too high for the following characterization aimed at evaluating the impact of the inhibition of WNT signaling in enhancing the killing ability of DCs through the activation of the immune system.

Our robust in silico interrogation highlighted the WNT pivotal role in nodal invasive behavior. Moreover, WNT-β-catenin axis is reported to drive invasive phenotype [16,17]. Subsequently, we conducted a functional validation assays to assess the impact of WNT-β-catenin targeting in vitro (Figure 6b). Of note, treatment with XAV-939 efficiently reduced the PDAC cell migration capacity (scratch assay, CTRL: 49.11 ± 1.53% versus XAV-939: 18.56 ± 1.26%-PANC-1. CTRL: 45.78 ± 0.46% versus XAV-939: 20.22 ± 0.52%-MIAPaca2; *p*-value < 0.0001); this result was not deemed related to the cell proliferation rate (Supplementary Figure S2). Next, we further analyzed the impact of WNT-β-catenin targeting invasive phenotype showed that only in PANC-1 model, XAV-939 strongly reduced the cell invasiveness (Figure 6c).

#### 2.4.2. XAV-939 Induced Modulation of β-catenin Expression

**Figure 7:**
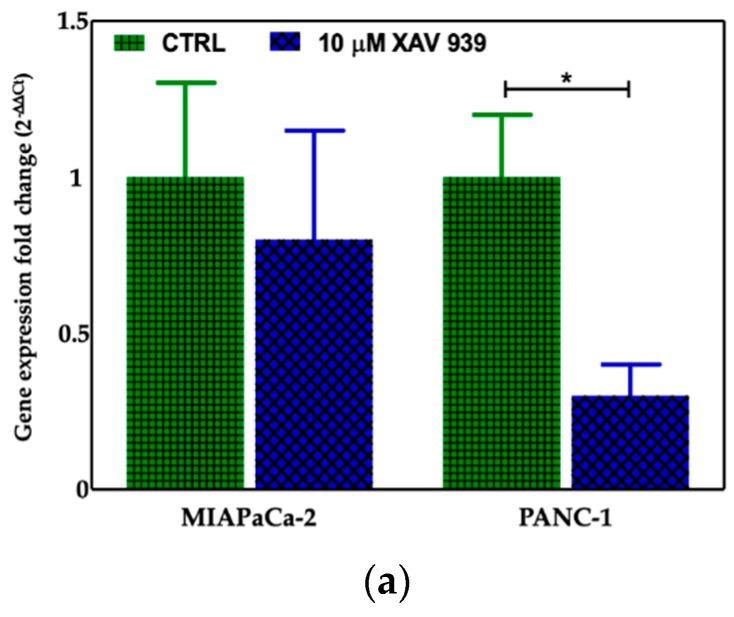

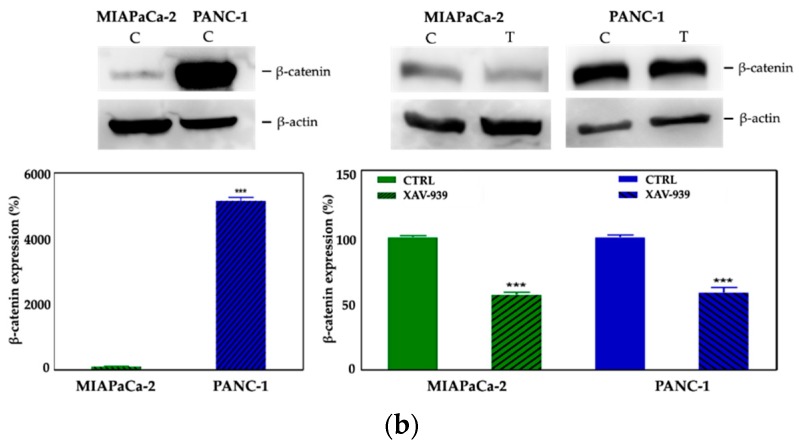
(**a**) Evaluation of β-catenin expression in PDAC cells treated with XAV-939 (10 μM) for 48 h by quantitative PCR (qPCR), * *p*-value < 0.05. (**b**) Evaluation of β-catenin expression in PDAC cells treated with 10 μM XAV-939 for 24 h by Western blotting and analysis of the bands by Image Lab software. C: control cells; T: 10 µM XAV-939 treated cells. β-actin was used to normalize protein expression levels. *** *p*-value < 0.001.

We selected 10 μM as the suitable XAV-939 concentration for the killing experiments because it was reported as the IC50 after 5 days in colony formation experiments [18]. To confirm the drug activity on the modulation of WNT-β-catenin pathway, we determined its ability to inhibit β-catenin. Thus, we investigated both the β-catenin-mRNA and protein expression in both PDAC cell lines. The levels of the mRNAs were normalized to the levels of glyceraldehyde-3-phosphate dehydrogenase (GAPDH) mRNA and reported as the ratio of mRNA copy number for the gene of interest to mRNA copy number for GAPDH (Figure 7a). We observed a reduction of the β-catenin-mRNA expression in 10 μM XAV-939-treated cells referred to control one, but only in PANC-1 cells the difference of expression was statistically significant (*p*-value < 0.05). The analysis of β-catenin expression by Western Blotting showed a higher expression of the protein in PANC-1 control cells than in MIAPaCa-2 ones and, upon XAV-939 treatment, we observed the reduction of β-catenin in both cell lines (Figure 7b), thus confirming the effect of XAV-939 on this specific pathway.

#### 2.4.3. XAV-939 Enhances Immune Cell-Dependent PDAC Cell Killing

**Figure 8:**
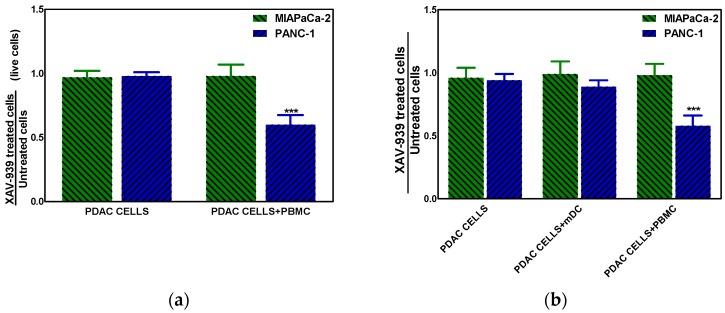
(**a**) Killing of PDAC cells in presence of 10 µM XAV-939 and PBMC. (**b**) Effect on cell proliferation by XAV-939 (10 µM) on PDAC cells in presence and absence of mDCs alone or PBMCs comprising mDCs. Results are expressed as fold change (*** *p*-value < 0.001 for PANC-1 treated cells vs. untreated ones).

The effect of XAV-939 on PDAC cells in the presence of complete PBMC comprising mDCs or mDCs alone was evaluated using a transwell insert in order to mimic the existing interaction between immune system and tumor in vivo and in order to evaluate the role of mDC as direct cytotoxic effectors against tumors, respectively. The mDCs were purified from PBMC as described in M&M section with a recover of about 5%. To this purpose the co-culture of PDAC cells and PBMC (ratio 1:100) or mDC (ratio 1:10) was treated with 10 µM XAV-939 for 48 h and results showed that only in the N^pos^ in vitro model, the killing of tumor cells in presence of PBMC plus XAV-939 was of 30.2 ± 2.1% whereas the antiproliferative effect was of 34.8 ± 1.9% (Figure 8a). Moreover, in presence of mDC we found a slight reduction (about 10%) of PANC-1 cells viability (Figure 8b).

#### 2.4.4. XAV-939 Modulates CD40 Expression in mDCs upon PDAC Cell Co-Culture

**Figure 9:**
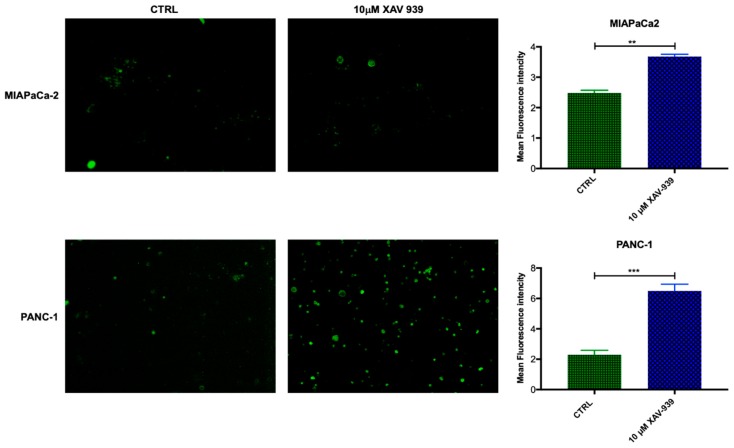
Immunofluorescent detection of CD40 in mDCs kept in contact with PDAC cells (MiaPaCa-2 in the upper panel and PANC-1 in the lower one) and XAV-939 (10 µM). The specimens were examined using a Leica (DMi8) immunofluorescence microscope. The images reported were recorded with 20× magnification and the quantification of the immunofluorescence was performed by ImageJ software.

In order to evaluate if mDC were activated by XAV-939, the increase in CD40 expression was investigated [19,20]. The mDCs, isolated from PBMCs, were kept in contact with the PDAC cells and whether they were treated or not with XAV-939 (10 µM), were stained with anti-CD40-FITC in order to investigate any difference in the activation of them upon treatment. As evidenced in Figure 9, mDCs resulted more activated when cells were in contact with PANC-1 and treated with XAV-939 compared to the condition without drug treatment, while mDCs did not display any different activation status between treated and control cells in the presence of MIAPaCa-2 cells.

### 2.5. Effect of XAV-939 on Short-Term Culture of PDAC Cells Cancer-Related Protein Profile

**Figure 10:**
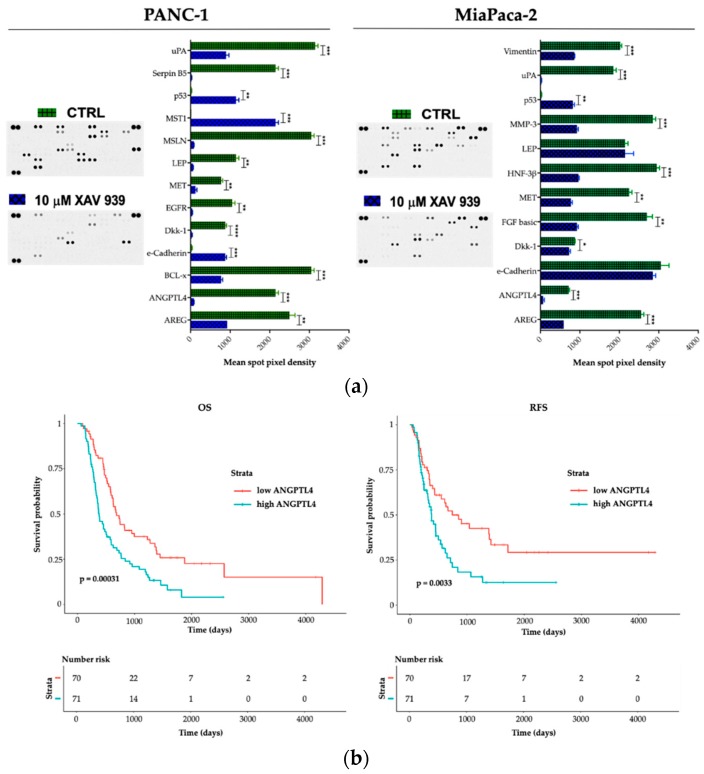
(**a**) A human XL oncology array of 84 human cancer-related protein was performed on PANC-1 and MIAPaCa-2 CM before and after treatment with XAV-939 (10 µM) for 48 h. Array spots were analyzed with ImageJ Lab 1.51 Software and normalized to positive control signal intensities. Graph bars represent the pixel density of the detected cancer-related proteins in two independent experiments. Values are expressed as mean ±SD of ten independent experiments. * *p* < 0.05; ** *p* < 0.05; and *** *p* < 0.001; **** *p* < 0.0001, versus control. (**b**) Survival function of ANGPTL4^high^ versus ANGPTL4^low^ PDAC patients.

To explore potential factors that differentiate PANC-1 and MIAPaCa-2 biological phenotype upon XAV-939 treatment and in order to deeper characterize the proteomic landscape that imply the PDAC invasive behavior, we compared cell lysates obtained from PDAC cells before and after XAV-939 exposure with a Human Proteome Oncology Profiler Array (Figure 10). XAV-939 strongly prevented cancer-related protein expression and increased pro-apoptotic and immunostimulating factors by PANC-1 such as amphiregulin (AREG), angiopoietin-like 4 (ANGPTL4), BCL2L1 (BCL-x), Dickkopf-1 (Dkk-1), ErbB (EGFR), HGF R/c-MET (MET), leptin (LEP), mesothelin (MSLN), Serpin B5, and urokinase (uPA) on PANC-1 cells (Figure 10a, left). Remarkably, e-Cadherin, p53, and MST1 protein expression was significantly enhanced upon XAV-939 exposure. Conversely, MIAPaCa-2 cells treatment resulted in significant oncologic protein expression modulation, impacting other relevant scatter factors such as FGF basic and MMP-3, confirming a strong modulation of the expression of several cancer-related genes (Figure 10a, right).

Therefore, we interrogated our cohort of 143 cases and identified a prognostic impact in terms of both OS and RFS for ANGPTL4 (Figure 10b), for OS regarding DKK-1 (DKK-1^high^ versus DKK-1^low^ * *p* < 0.05). We also found a trend FGF-2 and MST-1 impact on prognosis in terms of OS (FGF-2 high versus FGF-2 low *p* = 0.07 and MST-1 low versus MST-1 high *p* = 0.09). Finally, on an independent dataset from 176 pancreatic cancer patients we confirmed the prognostic role of the aforementioned genes (Supplementary Table S1).

## 3. Discussion

Lymph node metastatic process represents a validated prognostic factor and arose as an emergent unmet clinical need target, in order to better stratify the therapeutic strategy in PDAC patients [21,22]. Nodal dissemination results from the complex interaction between tumor intrinsic characteristics, such as invasiveness and migration potential [23] and immune microenvironmental features [24].

Interestingly, our bioinformatic analysis revealed WNT2 and WNT pathway, well described drivers of invasiveness, epithelial mesenchymal transition, drug-resistance and metastatic potential [25,26,27,28], as a significant signature enriched in N^pos^ patient group. Therefore, we investigated whether WNT was responsible for such an invasive biologic feature and demonstrated that specific blocking with XAV-939 significantly impaired tumor motility in two different cell lines. 

Moreover, the in silico deconvolution obtained from N^pos^ samples identified a substantial tumor tolerogenic immune microenvironment. Intriguingly, WNT signaling displays a central role in the immune-homeostasis against cancer [10,11,12]. The enrichment analysis let us to recognize an immune infiltrate constituted by enriched aDCs, HSCs and tolerant macrophages (M2) (Figure 5), providing novel translational frontiers on top of the existing data [29,30,31,32,33] within the node-metastatic clinical setting of PDAC. We identified a peculiar aspect of N^pos^ PDAC tumor propensity in educating the immune surveillance, reducing effector CD8 and CD4 T cells. Furthermore, we identified a cytokine basket that pinpoints the relationship between WNT2 overexpression and an immune-permissive tumor-environment. Interestingly, we describe a significantly enhanced immune-tolerogenic cytokine and chemokine pattern in N^pos^ patient’s subgroup; conversely, the tumor-suppressive cytokine profile resulted significantly downregulated within the N^pos^ patient’s subgroup (Figure 4b).

In vitro experiments validated the concept that WNT axis acts as one of the main causes of a vicious DCs-T cell-PDAC interaction. First, XAV-939 treatment is able to restrain the upregulation of the WNT/β-catenin in both DCs and PDAC cells, as demonstrated in other tumors [34,35]. Secondly, direct and microenvironment mediated DC-PDAC interactions can upregulate immunosuppressive WNT pathway [19,36,37]. Therefore, we employed XAV-939 triggering PBMC-mediated tumor cytotoxicity against PDAC cells. Remarkably, cell viability at the selected XAV-939 did not exert directed cytotoxic antitumoral effect (Figure 6a). Only upon co-culture with PBMCs we observed a CD40 upregulation upon treatment with XAV-939, highlighting a key tool in elicitation of an effective immune mediated anti-tumor response (Figure 9). It is interesting to highlight that PANC-1 and MIAPaCA-2 significantly differ from an anatomic-clinic and biologic perspective. PANC-1 cells are obtained from a patient with node metastases. Conversely, MIAPaCa-2 cells originated from a locally advanced patient without nodal involvement. Furthermore, XAV-939 efficacy in restoring a cancer-specific DC-dependent killing could be explained by two orders of evidences. First, MIAPaCa-2 expresses E-cadherin and CD44 while PANC-1 do not [38]. Secondly, XAV-939 specifically inhibits tankyrase PARP activity, dramatically decreasing DNA-PKcs protein levels [39], PARP3 controls TGF-β and ROS driven epithelial-to-mesenchymal transition and stemness by stimulating a TGF-β-Snail-E-cadherin axis [40]. Our data point out that tankyrase inhibition could have exerted a more pronounced effect on PANC-1 compared to MIAPaCa-2. Firstly, WNT inhibition restored MST-1 expression in PANC-1, contributing to the cancer suppression via ROS production [41,42]. Moreover, in M2 enriched PDAC patients (Figure 5), MST-1 induction towards WNT inhibition could represent a tool to circumvent the pro-tumor M2 addicted immune-suppressive environment [43]. Secondly, due to the differential E-cadherin expression, PANC-1 can be controlled in their invasive phenotype [44]. Conversely, the absence of E-cadherin modulation might contribute to the MIAPaCa-2 relative decreased sensitivity to XAV-939. Nonetheless, WNT inhibition in vitro showed its efficiency as oncogenic controller in both PDAC cell models. XAV-939 modulated several well-known oncogenic-related genes, partially restoring p53 expression and modulating uPA, MET and AREG expression [45]. Finally, ANGPTL4 resulted in a significant downregulation upon XAV-939 treatment, also showing clinical relevance as a negative prognostic factor in PDAC patients (Figure 10 and Supplementary Table S2). Interestingly, ANGPTL4 is strongly related to angiogenesis and tumor migration via α5β1-integrin/RAC1 interaction. Therefore, ANGPTL4 interrupts the interaction between cadherin-5 and claudin that leads to disruption of cell–cell adhesion, promoting metastatization [46,47]. ANGPTL4 is also involved in the regulation of immune homeostasis, modulating inflammatory T cell response and cytokines expression such as IL10 and IL1. These compelling evidences corroborated our in silico data (Figure 4b) being also reported as immune activation repressor [48]. Remarkably, our experimental evidences parallel previous report about ANGPLT4, as an upstream regulator of WNT pathway and an agonist of PKC, the main XAV-939 target [49]. Collectively, we propose our pilot transcriptomic and proteomic analyses as biologic baskets, potentially able to select PDAC phenotypes potentially druggable by WNT inhibition. WNT-spliceosome-related genetic signatures, evaluable in a multigene panel for pathway analysis, are already under active investigation in PDAC [50].

Clinical studies designed to translate these evidences into pre-clinical and clinical models are already ongoing [28].

These levels of data highlighted the tumor heterogeneity within different subsets of patients. The in silico and in vitro models embrace the potential to deeper characterize peculiar biological phenotypes in a specific PDAC subjects. N^pos^ patients with WNT overexpression along with immune suppressive microenvironment enrichment could display a more aggressive disease, representing potential candidates for an upfront neoadjuvant therapy. It is reasonable to speculate that WNT targeting in this clinical subset can exert therapeutic activity at two fundamental levels, addressing the invasive tumor behavior and re-educating an immune-reactive tumoral environment within the cancer milieu with an immune homeostatic loss.

Well designed, statistically powered studies are needed to better validate this hypothesis. 

WNT pathway and WNT2 in particular appear to coordinate a peculiar invasive behavior that enhances tumor invasiveness, being, at the same time, a pivotal driver for immune escape in N^pos^ PDAC.

## 4. Material and Methods

### 4.1. Dataset

The PACA-CA project cohort was selected from ICGC data portal (https://dcc.icgc.org/), due to availability of RNA-Seq. The selection of the cohort on the basis of a pathological stage allowed to obtain a subset with a good sample size (n = 143), suitable to perform statistical analyses.

### 4.2. Differential Gene Expression Analysis, GO Enrichment, and Biological Network

A raw read count matrix was used to identify significantly deregulated genes (DEGs) by DESeq2 R package [51]. Results were considered significant when adjusted *p*-value < 0.05 was obtained. A TopGO R package [52]. was used to perform gene ontology (GO) biological process enrichment analysis and to depict GO subgraph including the 10 most significant GO terms. The entire list of DEGs was used to build up a biological network of enriched terms of GO biological process, GO immune system process, KEGG, and Reactome pathway with ClueGO Cytoscape (3.7.1 version) app (v2.5.3) [53]. GSEA java application [54] was used to identify enriched gene sets of the preranked DEG list, considering results significant when FDR q-value < 0.25 was detected.

### 4.3. In silico Estimation of Microenvironmental Cell Types

A normalized expression matrix was downloaded to perform deconvolution and subsequently an estimation of microenvironment composition [55]. Enrichment scores of the different cell types were categorized on the basis of median values to perform chi-square Fisher test. Survival analyses has been performed by the “survival” R package.

### 4.4. Cell Culture

PANC-1 and MIAPaCa-2pancreas ductal adenocarcinoma (PDAC) cell lines were purchased from ATCC^®^ and grown as recommended. PBMC were obtained from volunteer blood donors according to the Declaration of Helsinki. This research has been approved by IRCCS Cancer Institute “Giovanni Paolo II” of Bari ethic committee on March 2016 (ethic code n. 574). Myeloid dendritic cells (mDC) were isolated from PBMC by means of CD1c (BDCA-1)^+^ Dendritic Cell Isolation Kit (MiltenyiBiotecGmbH, Friedrich-Ebert-Straße 68, 51429 Bergisch Gladbach, Germany) according to manufacturer’s instructions. All materials for cell culturing were purchased from EuroClone, Italy.

### 4.5. β-Catenin Expression by Real-Time PCR

To evaluate the effect of XAV-939 on mRNA expression of β-catenin, PDAC cells were treated with the drug (10 μM) for 48 h. The treated cells were then harvested, total RNA was extracted by means of QIAzol^®^ Lysis Reagent (QIAGEN Sciences, Maryland 20874, USA), and used for synthesis of cDNA with High Capacity cDNA Reverse Transcription Kit (Applied Biosystems™, Thermo Fischer Scientific Baltics UAB-Lithuania, Vilnius, Lithuania). The relative expression of β-catenin was measured in a StepOnePlus™ Real-Time PCR System (Applied Biosystems™-USA) using specific primers for the gene and PowerUp™ SYBR™ Green Master Mix mRNA quantitative real-time polymerase chain reaction Kit (Applied Biosystems™-Austin, TX, USA). Forward (FW) and reverse (RV) specific primer sequences for β-catenin gene are 5′ GCT GGG ACC TTG CAT AAC CT 3′ and 5′ AAG CAT TTT CAC CAG GGC AG 3′, respectively. Gene expression was normalized to the level of GAPDH within each sample using the relative ΔΔCT method.

### 4.6. Cytoxicity Assay

The cytotoxicity of XAV-939 (Selleckchem-Karl-Schmid-Str. 14, 81829 Munich, Germany) on PANC-1 and MIAPaCa-2 cell line was investigated, after exposure for 48 h to different concentrations of the drug (in the range of 0.001 to 100 µM), using the Cell Counting Kit-8 (CCK-8, Enzo Life Sciences Inc., 10 Executive Boulevard Farmingdale, New York, 11735, USA) as previously described [56]. The results are shown as cell proliferation (%)/dose plots of the mean of three different experiments and IC_50_ values were calculated from dose-response curves using CalcuSyn software.

### 4.7. Killing Assay

PANC-1 and MIAPaCa-2 cells were seeded at a density of 100,000 cell/well onto polyester 12-well transwell inserts (pore size 0.4 µm, 12 mm diameter, and apical volume 0.5 mL), with PBMC seeded in the basolateral volume (1.5 mL) at a density of 10,000,000/well. As control, specimens PDAC cells were seeded alone onto polyester transwell insert. After 24 h from seeding the specimens, as described above were exposed to XAV-939 (10 µM) for 48 h, then cell viability of PDAC cells was evaluated with two different assays, i.e., CCK-8 for cell proliferation analysis and the Trypan blue exclusion test for cell viability evaluation. In order to determine PDAC cells proliferation avoiding cancer cells mixing with mDCs/PBMCs, the transwell inserts were replaced into a clean plate filled with medium supplemented with CCK-8 [56]. Alternatively, to assess the cytotoxic effect, the polyester discs were removed from the transwell inserts, washed twice with PBS 1X and transferred to a new well plate filled with 0.25% Trypsin-EDTA solution to allow PDAC cells recovering. Trypan blue dye solution was added to cells suspension to obtain a 1 to 2 dilution; then the % of live cells was determined through counting by TC20 TM Cell Counter (BIO-RAD). Results are shown as fold change of treated PDAC cells vs. untreated ones in presence or not of PBMC.

### 4.8. CD40 Expression in mDC 

The mDCs were isolated by the PBMCs, that had been in contact with PDAC cells and treated with XAV-939 or exposed to vehicle as control, using the proper isolation kit and stained with anti-Hu CD40-FITC (eBioscience^TM^, Carlsbed CA-USA). In particular, mDC were transferred to microcentrifuge for spinning and after a two-wash step in PBS 1X/FBS 3%, cells were incubated for 30 min at 4 °C with anti-Hu CD40-FITC (0.5 µg/test) diluted in 100 µL of PBS 1X/FBS 3%. Following one wash step in PBS 1X/FBS 3%, cells were resuspended in Vectashield (Vector Laboratories), dropped on slides and covered with coverslip for fluorescence microscope examination (Leica).

### 4.9. Migration Test by Wound Healing (Scratch) Assay

Confluent PDAC cells (PANC-1 and MIAPaca-2) on fibronectin (10 μg/mL)-coated (Sigma-Aldrich, Saint Louis, MO, USA) 6 cm^2^ dishes were scraped as a ‘wound’ with a pipette tip. Additional details are provided in previously published data [57,58].

### 4.10. Invasion Assay

PANC-1 and MIA PaCa-2 cell invasion was studied through Boyden chambers assay as previously described [37]. Briefly, PANC-1 and MIAPaCa-2 cells (2 × 10^4^) were placed in the upper well of the chamber, and the invasion assay was performed with or without 10 µM XAV-939 at 37 °C in 5% CO_2_. Images show the % cell invasion after XAV-939 treatment referred to control cells.

### 4.11. Western Blotting Analysis

After treatment of MIAPaCa-2 and PANC-1 cells for 24 h with XAV-939 (10 µM), the protein extracts of treated and untreated cells used as control, were obtained by homogenization in RIPA buffer and treated with 1 mM phenylmethylsulfonyl fluoride (PMSF). The protein levels of β-catenin were analyzed by WB as previously described [56] and β-actin protein levels were used to normalize the sample values. The blot detection was performed with ChemiDoc™ Imaging Systems and analyzed with the ImageLab software (Bio-Rad-USA). Antibodies: the monoclonal antibody β-catenin (D10A8) was provided by Cell Signaling-USA and anti-β-actin (AC-15), was provided by Sigma-Aldrich. A mouse-HRP and a rabbit-HRP (Bio-Rad Laboratories, USA) were used as secondary antibody. All experiments were performed in triplicate.

### 4.12. Proteome Profiler Antibodies Array

The effect of XAV-939 (Selleckchem-Karl-Schmid-Str. 14, 81829 Munich, Germany) on PANC-1 and MIAPaCa-2 cell line was investigated, after exposure for 48 h to different concentrations of the drug (in the range of 0.001 to 100 µM) and analyzed on cell lysates by Human XL Oncology Array kit (R&D Systems^®^, Minneapolis, MN, USA) according to the manufacturer’s instructions. Spots were quantified with ImageJ 5.1 Software (Bio-Rad) and values were reported as mean pixel density.

### 4.13. Statistical Analyses

Statistical significance has been calculated using two-way analysis of variance (ANOVA) followed by the Bonferroni post hoc tests and 2-sided Student’s *t*-test (GraphPad Prism Software Inc., San Diego, CA, USA, vers. 5). Data were indicated with * *p*-value < 0.05, ** *p*-value < 0.01, and *** *p*-value < 0.001. In silico survival estimation on the top de-regulated gene encoding protein obtained from the proteome profiler antibodies array, was carried out on the discovery cohort of 143 cases from the PACA-CA study and on validation 176 subjects dataset obtained from RNA-seq analysis available from Human Protein Atlas (www.proteinatlas.org) [45,59,60].

## 5. Conclusions

The in silico gene-expression analysis from 143 PDAC analyzed in this pilot studies highlight WNT pathway as a close link between biological underlying signature of invasive disease and permissive immune-milieu in PDAC. A comprehensive bioinformatic deconvolution, revealing the main biological and microenvironmental drivers for the lymph node dissemination is corroborated by an in vitro the effectiveness of WNT targeting via the potent Tankyrase inhibitor XAV939. XAV939 treatment halts PDAC cells migration and enhances the immune response against the cancer cells, providing a potential theranostic window for patients suffering for nodal positive PDAC. Ancillary, we identify ANGPTL4 and MST1 as important regulators of PDAC invasiveness with a remarkable prognostic impact that can envision statistically powered clinical studies.

## Supplementary Materials

The following are available online at https://www.mdpi.com/2072-6694/11/7/942/s1, File S1: List of significant terms and the related genes involved in the KEGG, GO Biological Process, Reactome pathway and GO immune system process databases network enrichment showed in Figure 3a,b. Figure S1. Survival curves for OS and RFS comparing N^pos^ patients stratified according to aDC enrichments. Figure S2: The proliferation of PANC-1 and MIAPaca-2 after XAV-939 treatment was evaluated by MTT assay at 0, 24, and 48 h. Bars represent the mean ±SD, n = 5. Table S1. Gene Expression profile summary comparison between survival characteristics from 176 patients divided in high- and low-expressers from the TCGA dataset [45,59,60].
