# Supplementary material for: Gene Expression Comparison between the Lymph Node-Positive and -Negative Reveals a Peculiar Immune Microenvironment Signature and a Theranostic Role for WNT Targeting in Pancreatic Ductal Adenocarcinoma: A Pilot Study
Source: Cancers (Basel). 2019 Jul 4;11(7):942. doi: 10.3390/cancers11070942 (PMC6678707; doi:10.3390/cancers11070942)
Supplement: Supplementary file 1 [file cancers-11-00942-s001.pdf]

	GOTerm	% Associated Genes	P Value
1	organ induction	8.33	0.003
2	regulation of epithelial tube formation	9.09	0.003
3	cell proliferation in midbrain	18.18	7.49E-4
4	positive regulation of catecholamine secretion	16.66	8.96E-4
5	morphogenesis of an epithelial bud	15	5.53E-5
6	hepatocyte differentiation	13.04	8.52E-5
7	primary lung bud formation	40	1.38E-4
8	midbrain dopaminergic neuron differentiation	13.63	7.43E-5
9	Wnt signaling pathway involved in midbrain dopaminergic neuron differentiation	14.28	0.001
10	canonical Wnt signaling pathway involved in midbrain dopaminergic neuron differentiation	33.33	2.06E-4
11	neurological system process involved in regulation of systemic arterial blood pressure	11.11	0.002
12	negative regulation of systemic arterial blood pressure	10.52	0.002
13	cardiac chamber formation	16.66	8.96E-4
14	adult heart development	12.5	0.001
15	negative regulation of bone resorption	14.28	0.001
16	regulation of systemic arterial blood pressure by baroreceptor feedback	40	1.38E-4
17	regulation of systemic arterial blood pressure by carotid sinus baroreceptor feedback	66.66	4.16E-5
18	cardiac ventricle formation	20	6.14E-4
19	negative regulation of myotube differentiation	9.09	0.003
20	cardiac right ventricle morphogenesis	8.33	0.003
21	cardiac myofibril assembly	8.33	0.003
22	baroreceptor response to increased systemic arterial blood pressure	66.66	4.16E-5
23	negative regulation of TCF-dependent signaling by WNT ligand antagonists	13.33	0.001
24	VLDL assembly	40	1.38E-4
25	activation of protein kinase C activity	50	8.31E-5
26	neurological system process involved in regulation of systemic arterial blood pressure	11.11	0.002
27	positive regulation of keratinocyte proliferation	9.09	0.003
28	very-low-density lipoprotein particle assembly	9.09	0.003
29	regulation of response to food	9.52	0.002
30	organ induction	8.33	0.003
31	negative regulation of systemic arterial blood pressure	10.52	0.002
32	negative regulation of response to food	14.28	0.001
33	negative regulation of response to nutrient levels	10	0.002
34	negative regulation of keratinocyte differentiation	10	0.002
35	negative regulation of bone resorption	14.28	0.001
36	prostate glandular acinus development	14.28	0.001
37	regulation of intestinal lipid absorption	22.22	4.92E-4
38	regulation of epithelial tube formation	9.09	0.003
39	regulation of systemic arterial blood pressure by baroreceptor feedback	40	1.38-4
40	negative regulation of appetite	14.28	0.001
41	positive regulation of catecholamine secretion	16.66	8.96E-4
42	lipoprotein transport	8.69	0.003
43	regulation of fibroblast apoptotic process	11.11	0.002
44	regulation of systemic arterial blood pressure by carotid sinus baroreceptor feedback	66.66	4.16E-5
45	regulation of intestinal cholesterol absorption	22.22	4.928E-4
46	positive regulation of fibroblast apoptotic process	18.18	7.49E-4
47	glucagon secretion	14.28	0.001
48	regulation of glucagon secretion	15.38	0.001
49	positive regulation of non-canonical Wnt signaling pathway	12.5	0.001
50	baroreceptor response to increased systemic arterial blood pressure	66.66	4.167E-5
51	Cdc42 protein signal transduction	11.11	0.002

52	negative regulation of glucagon secretion	40	1.382E-4
53	regulation of Wnt signaling pathway, planar cell polarity pathway	11.11	0.002
54	regulation of Cdc42 protein signal transduction	15.38	0.001
55	negative regulation of osteoclast differentiation	12.5	6.07E-6
56	fat digestion and absorption	14.63	9.155E-9
57	vitamin digestion and absorption	8.33	0.003
58	scavenging of heme from plasma	23.07	1.41E-5
59	binding and Uptake of Ligands by Scavenger Receptors	9.52	1.836E-5
60	scavenging by Class B Receptors	33.33	2.068E-4
61	scavenging by Class A Receptors	10.52	0.002
62	VLDL assembly	40	1.382E-4
63	chylomicron assembly	16.66	3.983E-5
64	plasma lipoprotein assembly	16.66	3.983E-5
65	plasma lipoprotein remodeling	10	1.918E-4
66	chylomicron remodeling	22.22	4.928E-4
67	HDL remodeling	20	6.14E-4
68	activation of protein kinase C activity	50	8.31E-5
69	triglyceride-rich lipoprotein particle remodeling	11.76	0.001
70	high-density lipoprotein particle remodeling	9.09	0.003
71	chylomicron assembly	33.33	4.2E-6
72	very-low-density lipoprotein particle assembly	9.09	0.003
73	regulation of response to food	9.52	0.002
74	negative regulation of response to food	14.28	0.001
75	negative regulation of response to nutrient levels	10	0.002
76	cell-cell adhesion involved in gastrulation	13.33	0.001
77	regulation of cell-cell adhesion involved in gastrulation	14.28	0.001
78	regulation of intestinal lipid absorption	22.22	4.92E-4
79	intestinal cholesterol absorption	15	5.53E-5
80	negative regulation of appetite	14.28	0.001
81	chylomicron remodeling	15.38	0.001
82	lipoprotein transport	8.695652	0.003
83	regulation of intestinal cholesterol absorption	22.22	4.92E-4
84	negative regulation of heterotypic cell-cell adhesion	14.28	0.001
85	negative regulation of B cell proliferation	8	0.003
86	glucagon secretion	14.28	0.001
87	regulation of glucagon secretion	15.38	0.001
88	positive regulation of non-canonical Wnt signaling pathway	12.5	0.001
89	regulation of cytokine secretion involved in immune response	8.69	0.003
90	Cdc42 protein signal transduction	15.38	0.001
91	negative regulation of glucagon secretion	40	1.38E-4
92	regulation of Wnt signaling pathway, planar cell polarity pathway	11.11	0.002
93	negative regulation of cytokine secretion involved in immune response	8.69	0.003
94	regulation of Cdc42 protein signal transduction	15.38	0.001
95	negative regulation of calcium ion transport into cytosol	11.11	1.39E-4
96	detection of light stimulus involved in visual perception	11.11	0.002
97	regulation of the force of heart contraction	8.88	2.42E-5
98	the canonical retinoid cycle in rods (twilight vision)	13.04	8.52E-5
99	myosin filament assembly	14.28	0.001
100	striated muscle myosin thick filament assembly	15.38	0.001

File S1: List of significant terms and the related genes involved in the KEGG, GO Biological Process, Reactome pathway and GO immune system process databases network enrichment showed in Figure 3 (a) and (b).

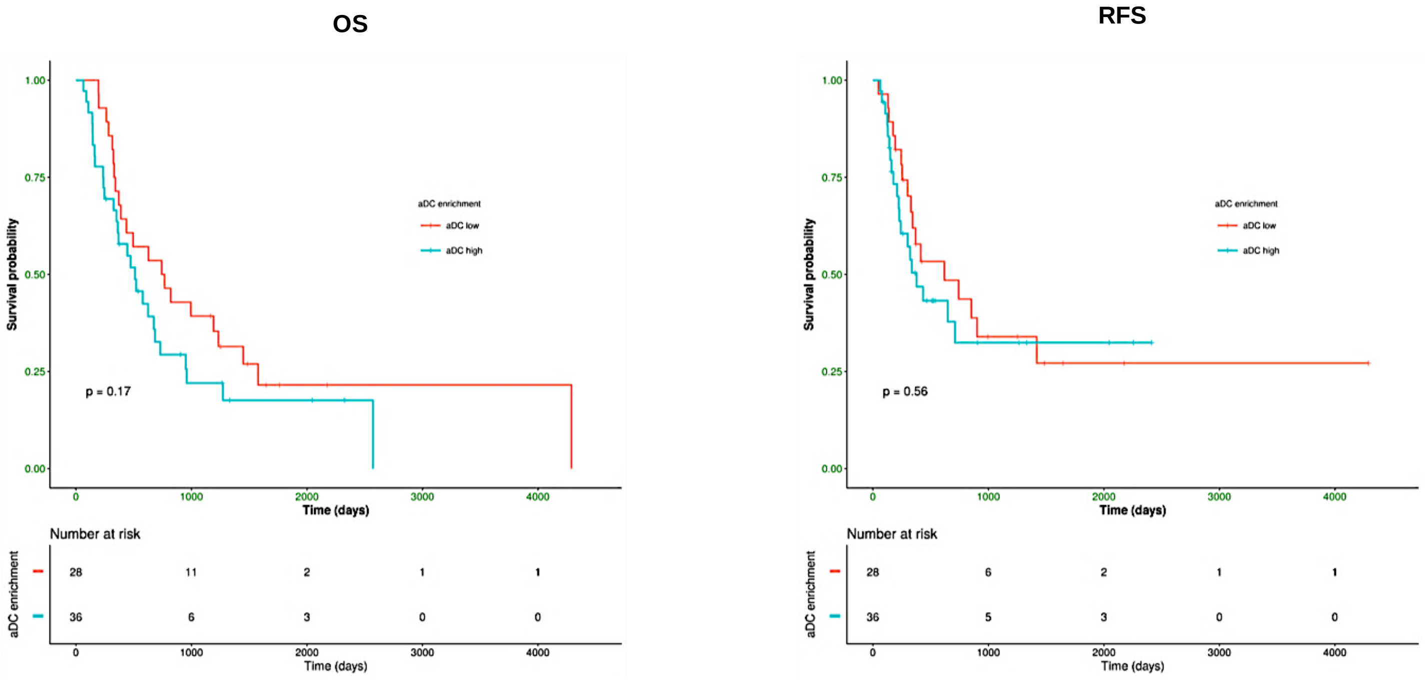

Supplementary Figure 1. Survival curves for OS (left panel) and RFS (right panel) comparing N^{pos} patients stratified according to aDC enrichments.

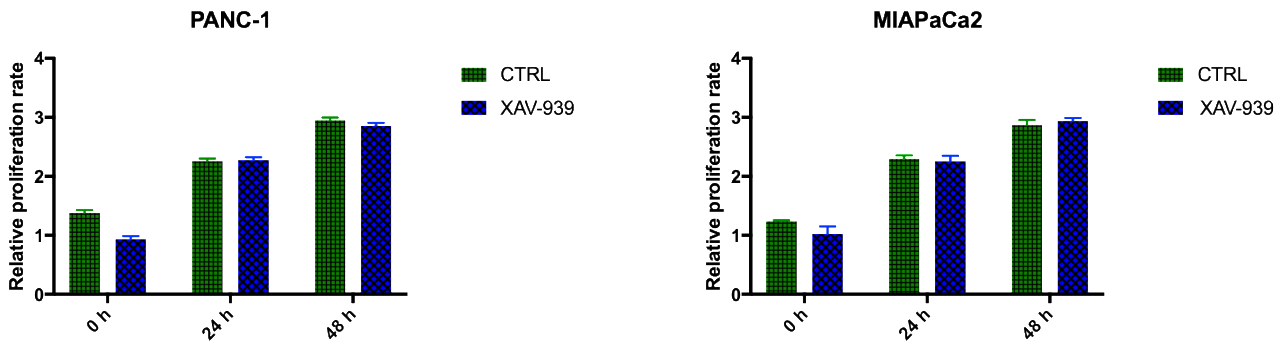

Supplementary Figure 2. The proliferation of PANC-1 and MIAPaca-2 after XAV-939 treatment was evaluated by MTT assay at 0, 24, 48 h. Bars represent the mean ± SD, n = 5.

	**Total cohort patients (n=176)**	
**Gene**	***P* score**	**5-years survival high vs low**
**WNT-2**	**0.014**	10% vs 36% (n=133 vs 43)
**ANGPTL4**	**0.0097**	20% vs 53% (n=137 vs 39)
**Dkk-1**	**0.00013**	19% vs 57% (n=135 vs 41)
**FGF basic**	**0.00098**	9% vs 36% (n=48 vs 128)
**MST1**	**0.00071**	53% vs 12% (n=49 vs 127)

Supplementary Table 1. Gene Expression profile summary comparison between survival characteristics from 176 patients divided in high- and low-expressers from TCGA dataset [45,59,60].
